# STAT3 signaling is associated with neuroimmune dysregulation in a Dravet syndrome model and pediatric drug-resistant epilepsy

**DOI:** 10.3389/fnins.2026.1810088

**Published:** 2026-04-13

**Authors:** Qi Zhang, Ji-Chen Wang, Hai-Qing Zhao, Jia Wang, Bing-Jie Xu, Li-Ping Zou

**Affiliations:** 1 Senior Department of Pediatric Medicine, Chinese PLA General Hospital, Beijing, China; 2 Medical School of Chinese PLA, Beijing, China; 3 Department of Urology, Chinese PLA General Hospital, Beijing, China; 4 School of Medicine, Nankai University, Tianjin, China; 5 Department of Pediatric Medicine, Hainan Hospital of Chinese PLA General Hospital, Beijing, China

**Keywords:** Dravet syndrome, drug-resistant epilepsy, immune dysregulation, inflammation, neuroinflammation, STAT3 signaling

## Abstract

**Background:**

Dravet syndrome (DS) is a severe developmental and epileptic encephalopathy, mainly caused by *SCN1A* gene mutations. Its core characteristics are heat sensitivity and refractoriness, and immunoinflammatory factors can participate in the occurrence and development of the disease. At present, the regulatory role of immune inflammation activation in DS has been confirmed, but the specific molecular core connecting systemic inflammation and central nervous system signals and its translational relevance to broader pediatric drug-resistant epilepsy (DRE) remains unclear.

**Methods:**

We conducted a multi-level integrative analysis combining transcriptomic mining of the GEO database to identify the *Stat3* hub, with clinical validation in a real-world pediatric cohort from our hospital, comparing DRE (including DS) and self-limited epilepsy with centrotemporal spikes (SeLECTS), to assess the clinical relevance of systemic inflammatory indices (NLR, SII, CRP). Findings were mechanistically verified in *Scn1a*^+/−^ mice via qRT-PCR, Western blotting, and immunofluorescence, using robust linear models to confirm central-peripheral inflammation correlations.

**Results:**

Transcriptomic profiling of *Scn1a*^+/−^ mice revealed a distinct inflammatory landscape (PC1 = 86%) dominated by JAK-STAT signaling, with *Stat3* identified as a consensus hub. Clinically, this systemic inflammatory signature was observed in our pediatric cohort (*n* = 140). Baseline inflammatory indices (NLR, SII, CRP) were significantly elevated in patients with drug-resistant epilepsy compared to those with SeLECTS (*p* < 0.001). Multivariable analysis further identified CRP as an independent factor closely associated with progression to drug resistance (OR = 2.79, *p* = 0.025). *In vivo* validation confirmed p-STAT3 hyperactivation in hippocampal gliosis (*p* < 0.0001), which exhibited robust linear correlations with peripheral markers (*r* ≥ 0.94, *p* < 0.001).

**Conclusion:**

This study identifies systemic and neuroinflammatory changes in DS associated with increased STAT3 signaling and this inflammatory signature is also observed in the broader pediatric DRE population. By bridging verified molecular mechanisms with real-world clinical data from our pediatric cohort, we suggest peripheral indices (NLR, SII, CRP) that may serve as accessible clinical indicators of disease severity in pediatric DRE. Pending functional validation, these findings identify STAT3 as a pathway of interest and a potential therapeutic candidate, supporting the development of adjunctive anti-inflammatory therapies targeting neuroimmune cascades for DS and broader refractory epilepsies.

## 1 Introduction

Epilepsy affects approximately 1% of the global population, with *SCN1A* mutations identified as a predominant genetic driver (Xie et al., 2020; GBD Epilepsy Collaborators, 2025), accounting for 38% of monogenic cases in our prior cohort (ChiCTR1900022164) (Wang et al., 2025). The most severe manifestation of these mutations is Dravet syndrome (DS), a catastrophic infantile-onset developmental and epileptic encephalopathy (Peycheva et al., 2020). While *SCN1A* haploinsufficiency and subsequent NaV1.1-dependent interneuron failure are primary etiologies, genotypic uniformity often fails to predict the substantial clinical heterogeneity in DS (Zhang et al., 2025). This divergence implicates pathogenic drivers beyond the ion channel itself, redirecting focus toward malleable pathways that intersect with network excitability.

Emerging frameworks position neuroinflammation not merely as a reactive consequence, but as an active propellant of epileptogenesis. In Dravet models, seizure recurrence correlates with aberrant hippocampal inflammation and blood–brain barrier compromise, creating a permissive environment for immune cell infiltration (Zhao et al., 2022). These inflammatory cascades likely sustain a pro-excitatory milieu rather than simply accompanying neural activity (Brenet et al., 2024). Within this landscape, the JAK2/STAT3 signaling axis emerges as a critical integration node, coupling cytokine stimulation with transcriptional programs that perpetuate glial activation and circuit vulnerability.

However, the precise architecture of STAT3 signaling within the *SCN1A*-deficient, fever-sensitive brain remains opaque. Although STAT3 is an established immunomodulator, its specific function as a network hub bridging neuroimmune activation and seizure susceptibility in DS has not been systematically delineated (Zimmerman et al., 2024). It remains unclear whether these accessible systemic markers can serve as reliable mirrors of the central STAT3-driven pathology. This necessitates a rigorous interrogation of the STAT3 pathway to determine its role as both a mechanistic key regulatory node and a translational target.

Here, we interrogate the STAT3-centered regulatory landscape in DS through a convergent bioinformatics and experimental framework. By integrating transcriptomic data from Gene Expression Omnibus (GEO) transcriptomes with clinical validation from a real-world pediatric cohort of drug-resistant epilepsy (DRE) and self-limited epilepsy with centrotemporal spikes (SeLECTS, a common age-dependent focal epilepsy with favorable outcomes serving as the clinical baseline control), we identify STAT3 as a signaling component associated with secondary neuroinflammation and evaluate its association with accessible systemic surrogates. Crucially, we functionally verify these findings in *Scn1a*^+/−^ mice. This integrative strategy provides biological context for the observed correlations, decoding the intersection of STAT3-mediated secondary inflammation and *SCN1A* deficiency to evaluate these systemic markers as reliable mirrors of central pathology.

## 2 Materials and methods

### 2.1 Study design and workflow

**Figure 1:**
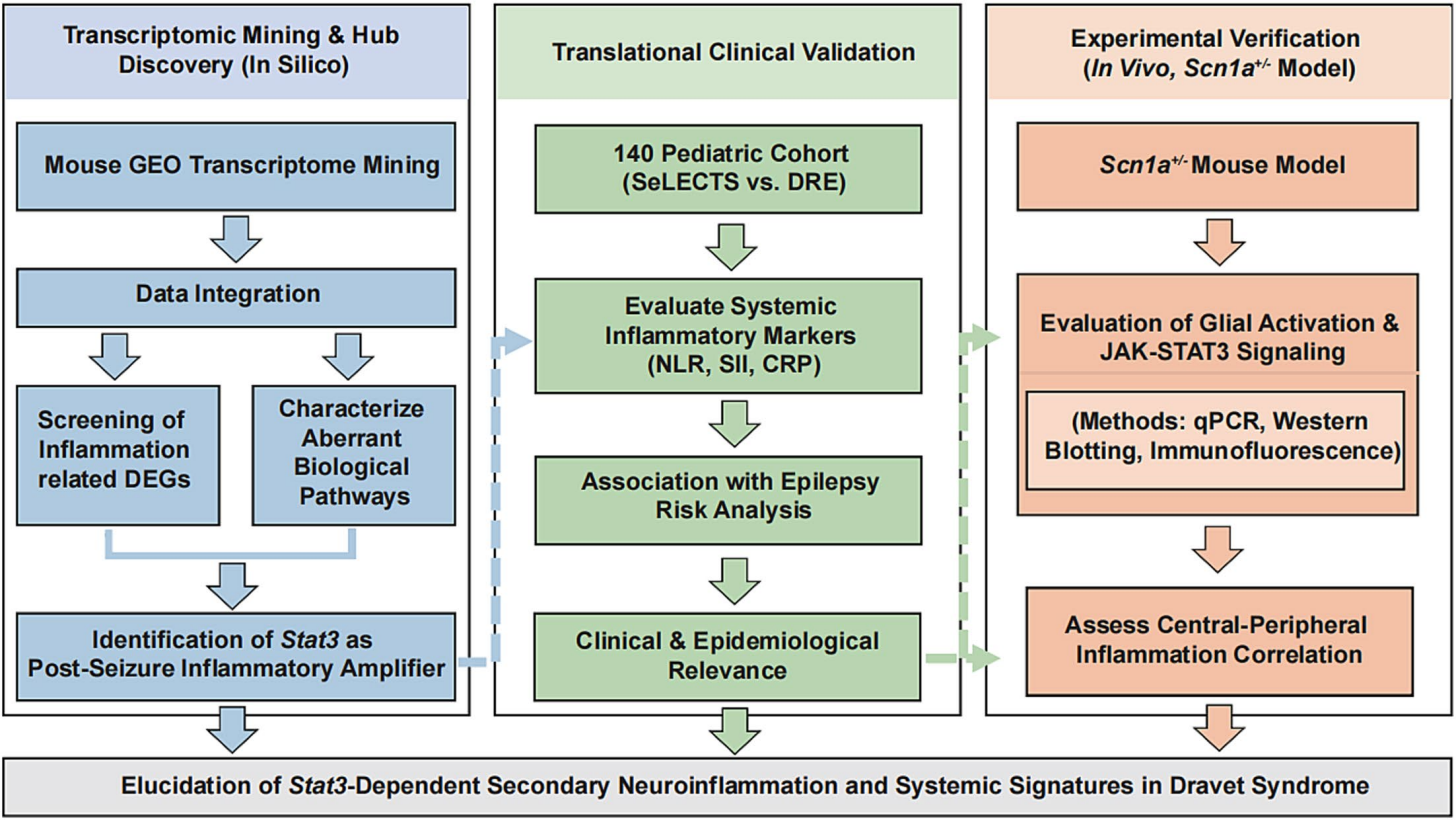
Flowchart of the study design. The workflow integrates transcriptomic discovery of inflammatory drivers, clinical validation of systemic markers (NLR, SII, CRP) in a pediatric clinical cohort, and *in vivo* verification of the JAK-STAT3 axis and glial activation in *Scn1a*^+/−^ mice.

This study was conducted as a multi-level integrative analysis to investigate the inflammatory and immune mechanisms underlying DS. Initially, a transcriptomic dataset from the Gene Expression Omnibus (GEO) was integrated to identify inflammation-related differentially expressed genes (DEGs) and screen for core upstream transcription factors (TFs). To establish the clinical and epidemiological relevance of these molecular findings, systemic inflammatory markers including the neutrophil-to-lymphocyte ratio (NLR), systemic immune-inflammation index (SII), and C-reactive protein (CRP) were evaluated for their association with epilepsy risk using a clinically matched pediatric cohort from our institution. Furthermore, the molecular and clinical evidence was verified through *in vivo* experiments using a *Scn1a*^+/−^ mouse model, which involved characterizing neuroinflammatory pathology in the hippocampus and cortex via quantitative PCR, Western blotting, and immunofluorescence, as well as assessing the correlation between central neuroinflammation and peripheral inflammatory profiles. The overall workflow, encompassing data integration, population-level validation, and experimental verification, is illustrated in Figure 1.

### 2.2 Data acquisition and processing

Mining public transcriptomic datasets from the GEO database has proven to be a highly effective and reliable strategy for elucidating core molecular mechanisms and identifying novel therapeutic targets across various central nervous system (CNS) disorders (Zha, 2025). Raw RNA-sequencing data were retrieved from the GEO database under accession number GSE112627. Concurrently, an independent transcriptomic dataset (GSE289689) was incorporated for supplementary support, with its detailed bioinformatic workflow available in the Supplementary Methods. The GSE112627 dataset comprised six samples (Supplementary Table S1), with gene-level quantification performed using HTSeq-count. Raw count data were assembled into a global expression matrix using R software (v4.4.1). Prior to differential expression analysis, low-abundance genes (defined as having a count <10 in more than 50% of samples) were filtered out to reduce noise and improve statistical power.

### 2.3 Differential expression analysis

Differential gene expression analysis between *Scn1a*^+/−^ and wild-type (WT) mice was performed using the DESeq2 package (v1.44.0). To ensure statistical robustness, *p*-values were adjusted using the Benjamini–Hochberg procedure to control the false discovery rate (FDR). Significant differentially expressed genes (DEGs) were defined by an adjusted *p*-value <0.05 and an absolute log_2_ fold change (|log_2_FC|) >0.585 (corresponding to a 1.5-fold change).

For dimensionality reduction and quality assessment, raw counts were normalized using the variance stabilizing transformation (VST). Principal component analysis (PCA) and uniform manifold approximation and projection (UMAP, n_neighbors = 5) were utilized to visualize global sample clustering and distinct group separation, with 95% confidence ellipses added via the ggforce package to delineate group boundaries. To visualize the overall distribution of DEGs, volcano plots were generated using ggplot2. Additionally, hierarchical clustering analysis of the top 100 upregulated genes was visualized using the pheatmap package (scaled by row *Z*-score) to highlight distinct expression signatures.

### 2.4 Functional enrichment and transcription factor analysis

Gene Ontology (GO), biological process and Kyoto Encyclopedia of Genes and Genomes (KEGG) pathway enrichment analyses were performed using the clusterProfiler package (v4.12.6). For significantly upregulated genes, gene symbols were converted to Entrez IDs using org.Mm.eg.db (v3.21.0). An FDR threshold of <0.05 was applied to identify significantly enriched terms and pathways.

To identify potential upstream regulators, all significantly upregulated genes were subjected to TF enrichment analysis using the Enrichr web tool. Predictions were sourced from three curated libraries: “ENCODE and ChEA Consensus TFs from ChIP-X,” “TRRUST Transcription Factors 2019,” and “Transcription Factor PPIs.” For each library, candidates were ranked by *p*-value, and the top 20 TFs from each were intersected to identify robust consensus regulators.

### 2.5 Construction of transcriptomic surrogates for CNS immune activation

To conceptualize the central inflammatory microenvironment, gene-set-based transcriptomic surrogates were constructed using variance stabilizing transformation (VST) normalized values. These indices are designed to reflect broader CNS myeloid and lymphoid activation states. Canonical marker genes were aggregated to represent the transcriptomic signatures of myeloid/neutrophil-like activation (*Sneu: S100a9, Fcgr3, Csf3r*), lymphoid activation (*Slym: Lck, Zap70*), and platelet-related pathways (*Splt: Itga2b, Gng11*). The central transcriptomic surrogates (transcriptomic NLR and transcriptomic SII) were calculated mathematically homologous to peripheral indices, serving as proxies for the central neuroimmune burden.


$$\mathrm{NLR}:\mathrm{NLR}=\log_{2}((S_{\mathrm{neu}}+1)/(S_{\mathrm{lym}}+1))$$

$$\mathrm{SII}:\mathrm{SII}=\log_{2}(((S_{\mathrm{neu}}+1)\times (S_{\mathrm{plt}}+1))/(S_{\mathrm{lym}}+1))$$

The expression levels of *Stat3* and the CRP-surrogate *Ptx3* were normalized using the same VST scale.

### 2.6 Pediatric clinical cohort validation

To validate the clinical translatability of the identified inflammatory signatures specifically in the context of severe pediatric epilepsy, a retrospective clinical cohort analysis was conducted at our center. The analytical cohort comprised 140 pediatric patients hospitalized between 2010 and 2025, divided into a DRE (*n* = 69... and a SeLECTS control group (BPE, *n* = 71).The systematic screening and enrollment workflow for this pediatric cohort is delineated in the flowchart provided in Supplementary Figure S1.

To delineate the clinical cohort and isolate disease-associated immune signatures, we employed a retrospective longitudinal design with stringent gating criteria. Inclusion necessitated: (1) a definitive clinical diagnosis of DRE or SeLECTS adjudicated by pediatric epileptologists; (2) age ≤14 years; and (3) the availability of complete serological profiles (inclusive of total white blood cell, neutrophil, lymphocyte, and platelet counts, alongside C-reactive protein) acquired at the initial hospital presentation, prior to the initiation of long-term poly-pharmacotherapy. To mitigate confounding systemic variables, patients were excluded if they presented with: (1) total leukocyte counts exceeding age-calibrated physiological limits, or concurrent clinical evidence of acute systemic infections; (2) preexisting primary hematological or systemic autoimmune disorders; or (3) documented exposure to systemic glucocorticoids or adrenocorticotropic hormone (ACTH) within 4 weeks preceding serological sampling.

Systemic inflammatory indices (NLR, SII, and CRP) were derived from serological profiles obtained at the initial clinical presentation. This approach was utilized to evaluate the association between the early post-seizure peripheral inflammatory burden and the subsequent progression to drug resistance.

### 2.7 Animals and hyperthermia-induced seizure model

The animal study was reviewed and approved by the Institutional Animal Care and Use Committee (IACUC) of Beijing BioWork Technology Co., Ltd. (Approval No. BW-IACUC-2025-172). The *Scn1a^tm1Kea^* mouse line (generously provided by Prof. Long-Jun Wu, Rutgers University) was maintained on a 129S6/SvEvTac background. To generate the experimental F1 hybrid cohorts, heterozygous (*Scn1a*^+/−^) mice were crossed with C57BL/6J wild-type mice. Genotyping was performed via PCR to identify *Scn1a*^+/−^ mutants and wild-type (WT) littermates (Supplementary Figure S2C). The amplification utilized a three-primer system, including a common forward primer, a wild-type reverse primer, and a mutant-specific reverse primer, to distinguish the genotypes based on DNA fragment size. Detailed sequences for these genotyping primers are provided in Supplementary Table S2.

A total of 48 male F1 mice aged 3 weeks were assigned to the DS model group (*Scn1a*^+/−^, *n* = 24) and the control group (WT, *n* = 24). To assess seizure susceptibility, a hyperthermia-induced seizure protocol was employed. Briefly, core body temperature was elevated by 0.5 °C every 2 min using a controlled infrared heating lamp until a generalized seizure occurred or a threshold of 43 °C was reached. Seizure threshold temperature, duration, and severity (Racine scale) were recorded. Detailed housing conditions and induction procedures are provided in the Supplementary Methods.

### 2.8 Anesthesia, euthanasia, and tissue collection

To ensure animal welfare and experimental consistency, anesthesia and euthanasia were performed following a standardized protocol.

For terminal tissue harvesting, mice were deeply anesthetized via an intraperitoneal (i.p.) injection of the 1.25% (w/v) tribromoethanol solution at a dose of 250 mg/kg. The depth of anesthesia was confirmed by the loss of the pedal withdrawal reflex. Subsequently, mice were euthanized via transcardial perfusion with ice-cold phosphate-buffered saline (PBS) to remove systemic blood. The hippocampus and cortex were rapidly dissected on ice, snap-frozen in liquid nitrogen, and stored at −80 °C for molecular analysis. All brain tissues were harvested exactly 24 h after the hyperthermia-induced seizure to evaluate the acute-on-chronic neuroinflammatory response.

### 2.9 Peripheral inflammation assessment

Peripheral blood was collected from mice via retro-orbital puncture 3 days prior to euthanasia (which corresponds to 2 days prior to the hyperthermia-induced seizure protocol). This timeline was strictly established to capture the primary, genotype-driven baseline systemic inflammatory tone, excluding the acute physiological stress induced by hyperthermia. The NLR was determined from an automated complete blood count (CBC). Serum was separated by centrifugation (3,000 × g, 15 min, 4 °C) and stored at −80 °C for subsequent analysis of IL-6 and CRP levels using mouse-specific ELISA kits (ZC-137519, ZC-137382) per the manufacturer’s instructions.

### 2.10 Quantitative real-time PCR (qRT-PCR)

Total RNA was extracted from the hippocampus and cerebral cortex of *Scn1a*^+/−^ and WT mice using AG RNAex Pro RNA (Accurate Biology, AG21102). RNA (1 μg) was reverse transcribed to cDNA in a 20 μL reaction mixture using the qRT-PCR was performed with SYBR Green Pro Taq HS qPCR Kit III (Accurate Biology, AG11739) using QuantStudio 7 Flex (ABI). Primers for qRT-PCR are listed in Supplementary Table S2. Meltcurve analysis confirmed transcript-specific amplification. Gene expression was normalized to *GAPDH* as an internal control. Relative mRNA expression was calculated using the comparative cycle threshold method (CT), also known as 2^−ΔΔCT^ method.

### 2.11 Western blotting

Total protein was extracted from the hippocampus and cerebral cortex of *Scn1a*^+/−^ and WT mice using RIPA buffer (Cell Signaling Technology, 9806) supplemented with protease inhibitors and phosphatase inhibitors. Protein yield was determined with a BCA kit (Cell Signaling Technology, 7780). Equal lysate aliquots containing 20 μg of total protein per lane were separated by 10% SDS-PAGE and electrotransferred to PVDF membranes. Blocking procedures were optimized based on target characteristics. Membranes dedicated to phosphorylated targets were blocked with 5% bovine serum albumin (BSA) in TBST for 1 h at room temperature, whereas 5% non-fat milk was applied for non-phosphorylated proteins. The membranes were then incubated with primary antibodies overnight at 4 °C. The following antibodies were used: p-STAT3 (1:500, Affinity, AF3293), STAT3 (1:1000, Affinity, AF6294), IL-6 (1:1000, Affinity, DF6087), p-JAK1 (1:500, Affinity, AF2012), JAK1 (1:1000, Affinity, AF5012), Cyclin D1 (1:1000, Affinity, AF0931), GFAP (1:1000, Affinity, DF6040), IBA1 (1:1000, Affinity, DF6442), and GAPDH (1:5000, ZenBio, 200306-7E4). After washing with TBST, the membranes were incubated with HRP-conjugated species-specific secondary antibodies for 1 h at room temperature. To ensure rigorous quantitative normalization, membranes initially probed for phosphorylated kinases (p-STAT3 and p-JAK1) were stripped using a commercial stripping buffer, thoroughly washed, re-blocked, and reprobed for their respective total proteins and the loading control on the exact same membrane. Protein bands were visualized using the Tanon 4800 imaging system, and band intensity was quantified using ImageJ software.

### 2.12 Double immunofluorescence staining and Nissl staining

The hippocampus and cerebral cortex of *Scn1a*^+/−^ and WT mice were immersed in 10% neutral buffered formalin for 6 h, dehydrated in a gradient of ethanol, embedded in paraffin, and sectioned at a thickness of 5 μm. For double immunofluorescence staining, paraffin-embedded tissue sections were successively subjected to deparaffinization, initial antigen retrieval, peroxidase inactivation in regions of interest and permeabilization. Non-specific binding was blocked using 3% BSA at room temperature, followed by incubation with primary antibodies overnight at 4 °C. The primary antibodies used include NeuN (1:80, Affinity, DF6145), GFAP (1:500, Oasis Biofarm, OB-PGPO55-02), p-STAT3 (1:80, Affinity, AF3293), IBA1 (1:500, Oasis Biofarm, OB-PGPO49-02). Secondary antibodies include Goat Anti-Rabbit IgG H&L (AF488, ZenBio, 550037) and Donkey-anti-Guinea pig IgG (AF555, Oasis Biofarm, D-GP555). Following DAPI counterstaining and mounting, the sections were scanned and digitized using a ZEISS AxioScan7 digital slide scanner equipped with a 20× objective.

Paraffin sections were subjected to deparaffinization, and stained using the Nissl Stain Kit (Solarbio, G1430) per the manufacturer’s instructions. Slides were scanned on a digital pathology scanner (KF-PRO-120, KFbio).

### 2.13 Statistical analysis

Statistical analyses were performed using R software (version 4.4.1) for bioinformatics and clinical data validation, and GraphPad Prism (version 10.0) for experimental data visualization.

For comparisons between two groups, statistical significance was determined using the unpaired Student’s *t*-test for normally distributed data or the Mann–Whitney U test for non-normally distributed data.

To ensure statistical rigor, correlation analyses were tailored to the specific nature of the datasets. For the bioinformatics analysis of transcriptomic data, correlations were conducted using robust linear models (RLM) to mitigate the influence of potential biological outliers and background noise. Results for these transcriptomic analyses are reported as weighted correlation coefficients (weighted *r*) and *p*-values derived from robust *t*-tests. Conversely, for the *in vivo* experimental validation data (e.g., correlations between biochemical and histological quantitative assays), standard Pearson correlation analysis was utilized following the confirmation of normal data distribution.

For the pediatric clinical cohort, the normality of continuous variables was assessed using the Shapiro–Wilk test. Normally distributed data are presented as mean (standard deviation) and were compared using Student’s *t*-test. Skewed continuous variables, including hematological counts and systemic inflammatory indices (NLR, SII, CRP), are summarized as median (interquartile range, IQR) and were compared using the non-parametric Mann–Whitney *U* test. Categorical data are expressed as counts (percentages) and were analyzed via the chi-square test. To mitigate dimensional bias and facilitate direct effect size comparisons, continuous systemic inflammatory indices (NLR, SII, and CRP) underwent *Z*-score standardization prior to regression analysis. Multivariable logistic regression models were constructed to determine independent factors associated with the progression to drug resistance. To prevent multicollinearity, each standardized inflammatory marker was modeled separately, with all models adjusting for predefined covariates including age at onset, sex, and body mass index (BMI). Restricted cubic splines (RCS) coupled with logistic regression were applied to model the non-linear dose–response probabilities between inflammatory burden and the risk of refractory progression. Statistical significance was established at a two-sided *p* < 0.05.

## 3 Results

### 3.1 Transcriptomic profiling identifies *Stat3* as a hub regulator of the pro-inflammatory phenotype

**Figure 2:**
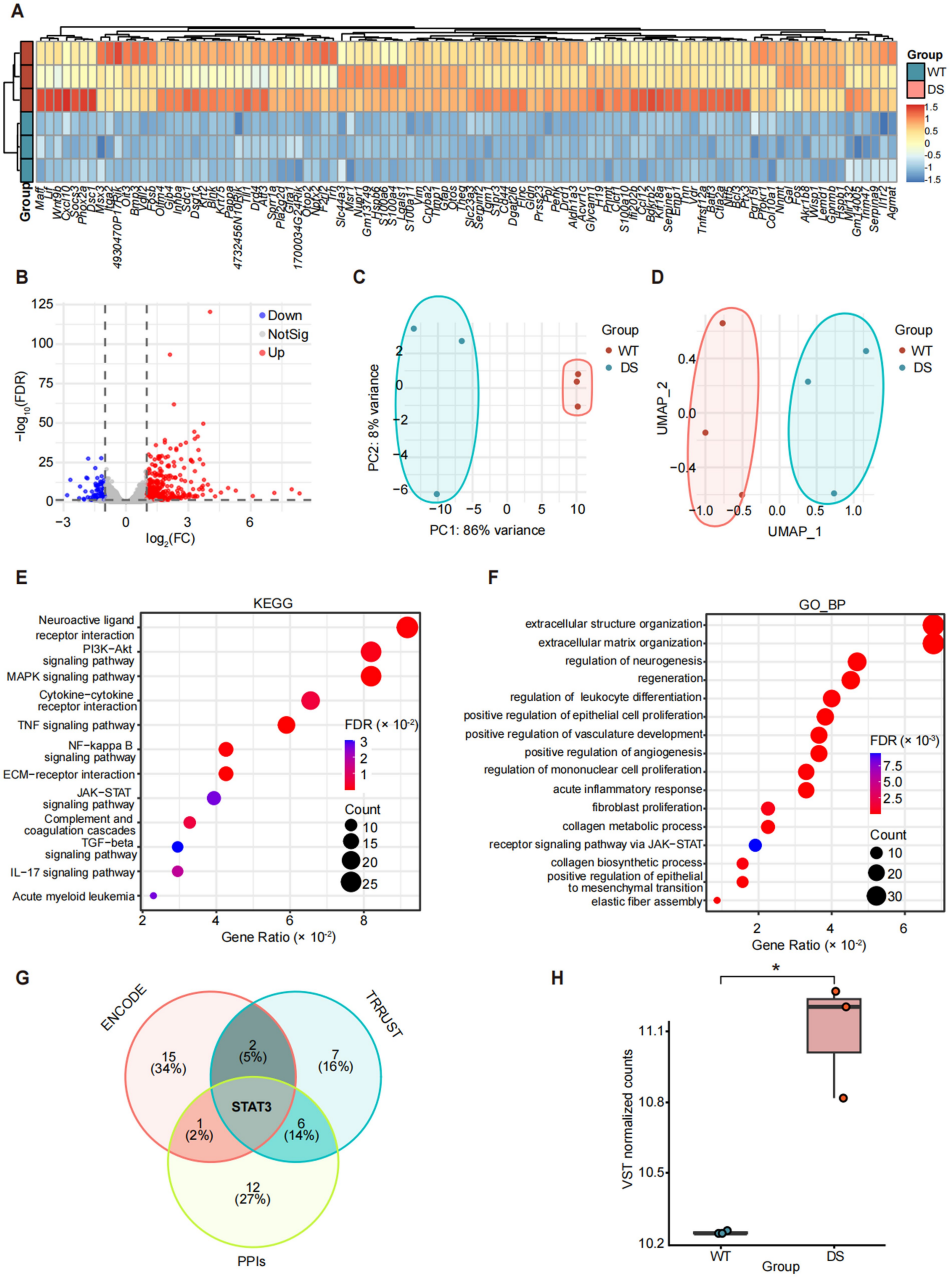
Transcriptomic landscape and consensus hub identification. **(A)** Hierarchical clustering heatmap of DEGs. **(B)** Volcano plot of significant DEGs. **(C,D)** Dimensionality reduction visualizing sample distribution via PCA **(C)** and UMAP **(D)** plots. **(E,F)** Functional enrichment analyses showing top KEGG pathways **(E)** and GO terms **(F)**. **(G)** Venn diagram of consensus TF predictions across three databases. **(H)** Box plot of brain Stat3 expression (VST).

To comprehensively delineate the molecular landscape associated with the post-seizure state in SCN1A deficiency, we analyzed bulk RNA sequencing data (GEO accession: GSE112627) derived from brain tissues of *Scn1a*^+/−^ (KO) mice and wild-type (WT) littermates on a [129S6 × B6] F1 background. Dimensionality reduction analyses confirmed distinct transcriptomic profiles driven by genotype. Principal component analysis (PCA) revealed a clear segregation of samples, with the first principal component (PC1) capturing 86% of the total variance, indicating that genotype is the primary driver of transcriptional heterogeneity (Figure 2C). This separation was further corroborated by the uniform manifold approximation and projection (UMAP) plot, which displayed two discrete clusters corresponding to the WT and KO groups (Figure 2D). Differential expression analysis identified a widespread dysregulation of the transcriptome in *Scn1a*^+/−^ mice. The volcano plot visualized the distribution of differentially expressed genes (DEGs), highlighting a significant number of up-regulated (red) and down-regulated (blue) genes based on stringent statistical thresholds (Figure 2B). To characterize the most prominent molecular shifts, we performed hierarchical clustering on the top 100 significantly up-regulated genes. The resulting heatmap demonstrated a robust and consistent elevation of these genes across all KO samples compared to controls (Figure 2A, top panel), suggesting a uniform pathological response to sodium channel haploinsufficiency.

To decode the biological significance of these transcriptional alterations, we conducted functional enrichment analyses. KEGG pathway analysis revealed a striking enrichment of inflammation- and immunity-related signaling cascades. The most significantly enriched pathways included the “TNF signaling pathway” (*p*_adj_ = 1.64 × 10^−6^) and “NF-kappa B signaling pathway” (*p*_adj_ = 3.38 × 10^−4^), indicating a severe inflammatory burden. Notably, pathways directly linked to STAT3 activation were also prominent, including “Cytokine-cytokine receptor interaction” (*p*_adj_ = 0.003) and the “JAK-STAT signaling pathway” (*p*_adj_ = 0.018) (Figure 2E).

Consistent with these findings, GO biological process analysis indicated a substantial activation of immune responses alongside structural remodeling. The top-enriched terms were dominated by “extracellular matrix organization” (*p*_adj_ = 2.02 × 10^−15^) and “acute inflammatory response” (*p*_adj_ = 2.69 × 10^−9^) (Figure 2F). Significantly the analysis identified the “receptor signaling pathway via JAK-STAT” (*p*_adj_ = 0.006) as a significantly enriched process. Detailed inspection of the gene lists within these inflammatory and signaling terms revealed the recurrence of Stat3 alongside other key mediators, collectively pointing to the JAK-STAT signaling axis as a potential key regulatory node of the neuroinflammatory phenotype in the *Scn1a*^+/−^ brain.

To identify the master regulators orchestrating this inflammatory program, we performed an upstream TF enrichment analysis using three independent databases: ENCODE, TRRUST, and transcription factor PPIs. Intersection analysis of predicted TFs from these sources identified STAT3 as the sole robust consensus hub regulator shared across all three independent datasets (Figure 2G). Furthermore, to validate this prediction at the transcriptional level, we quantified the specific expression of Stat3 in our RNA-seq data. As shown in the box plot, the normalized Stat3 expression was significantly elevated, rising from a median of approximately 10.25 (VST) in the WT controls to 11.21 (VST) in the DS group (Figure 2H). The centrality of this mechanism was further supported by an independent external supportive dataset (GSE289689), which demonstrated robust up-regulation of STAT3 expression and significant positive enrichment of the JAK-STAT signaling cascade in severe epilepsy phenotypes (Supplementary Figures S2A,B). Collectively, these data support an association between Stat3 and the observed inflammatory profile observed in *Scn1a*-deficient brains.

### 3.2 Central transcriptomic immune surrogates correlate with STAT3 expression

**Figure 3:**
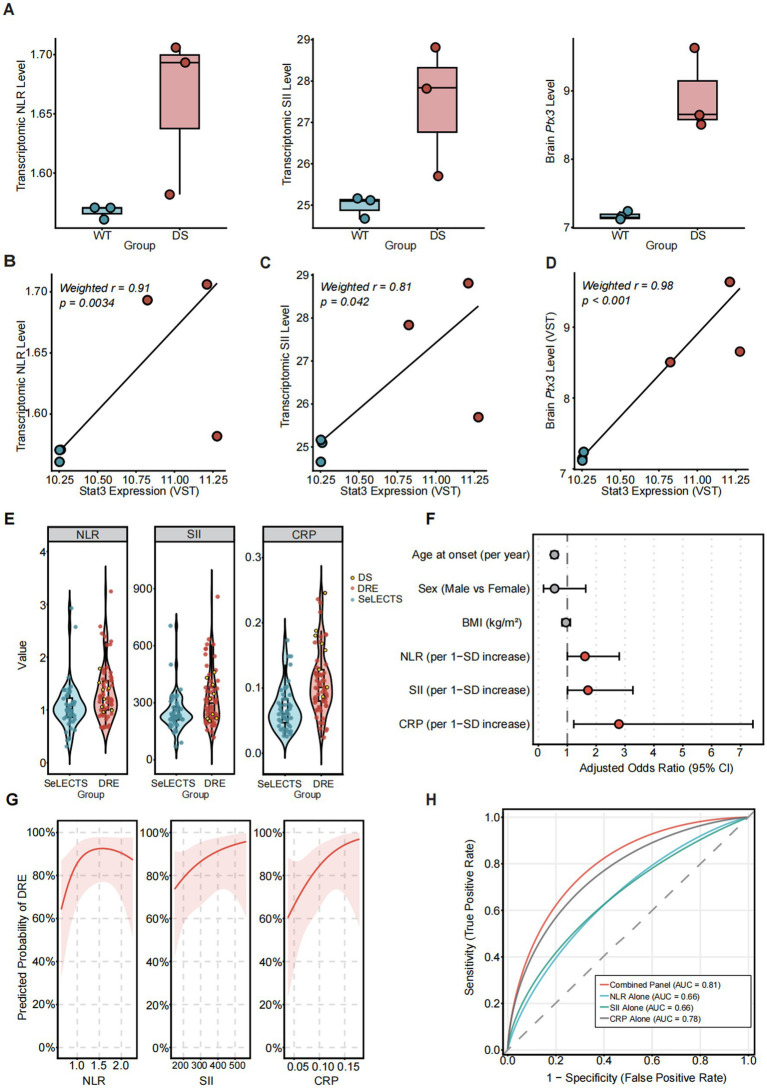
Systemic inflammatory profiles and clinical validation. **(A)** Box plots of transcriptomic markers (brain *Ptx3*, NLR, and SII) in WT and DS groups. **(B–D)** Correlation analyses between Stat3 expression (VST) and transcriptomic NLR **(B)**, SII **(C)**, and brain *Ptx3* **(D)**. **(E)** Violin plots of inflammatory markers in the SeLECTS and DRE groups. **(F)** Forest plot of multivariable logistic regression for DRE risk factors. **(G)** Dose–response curves of NLR, SII, and CRP for DRE probability. **(H)** Receiver operating characteristic (ROC) curves evaluating discriminative capacity.

To investigate the link between central pathology and systemic inflammation, we calculated composite inflammatory signatures using the specific marker gene summation method based on the DS transcriptomic datasets. Transcriptomic NLR, transcriptomic SII, and brain *Ptx3* levels were significantly elevated in the DS group compared to controls (Figure 3A). Robust correlation analysis revealed that the expression of the upstream hub *STAT3* was strongly and positively correlated with the transcriptomic NLR level (weighted *r* = 0.91, *p* = 0.0034; Figure 3B), the transcriptomic SII level (weighted *r* =0.81, *p* = 0.042; Figure 3C), and brain *Ptx3* levels (weighted *r* = 0.98, *p* < 0.001; Figure 3D). Collectively, these data suggest STAT3 as a signaling node associated with the observed neuroimmune changes.

### 3.3 Systemic inflammatory indices are elevated in DRE and the DS subgroup

To rigorously validate our findings in a clinically relevant context, we evaluated a pediatric cohort consisting of patients with DRE (*n* = 69, including 9 DS cases) and SeLECTS (*n* = 71) (Table 1). Baseline levels of NLR, SII, and serum CRP were significantly elevated in the DRE group compared to SeLECTS controls (all *p* < 0.001; Figure 3E). Multivariable logistic regression with *Z*-score standardization and adjustment for predefined covariates identified CRP as an independent factor associated with progression to drug resistance (OR = 2.79, 95% CI: 1.23–7.43, *p* = 0.025). Positive trends were observed for NLR (OR = 1.61, 95% CI: 1.00–2.80, *p* = 0.064) and SII (OR = 1.72, 95% CI: 1.01–3.27, *p* = 0.068) (Table 2 and Figure 3F). Restricted cubic spline modeling demonstrated a non-linear dose response trajectory where the probability of DRE escalated sharply as standardized inflammatory indices exceeded their respective thresholds (Figure 3G). Receiver operating characteristic (ROC) analysis evaluated the discriminative capacity of these markers, revealing moderate effect sizes. The combined inflammatory panel yielded the highest discriminative capacity with an area under the curve of 0.81, compared to single indicators including CRP (AUC = 0.78), SII (AUC = 0.66), and NLR (AUC = 0.66) (Figure 3H).

**Table 1:** Baseline demographic and laboratory characteristics of the study population, stratified by epilepsy status.

Variable	SeLECTS group (*n* = 71)	DRE group (*n* = 69)	DS subgroup (*n* = 9)	*p*-value
Age at onset, median [IQR], y	7.00 [5.00, 9.00]	1.00 [0.40, 3.00]	0.80 [0.40, 4.00]	<0.001
Sex, *n* (%)				0.283
Male	46 (64.8)	38 (55.1)	4 (44.4)	
Female	25 (35.2)	31 (44.9)	5 (55.6)	
BMI, median [IQR], kg/m^2^	17.12 [14.93, 21.30]	15.98 [14.82, 17.12]	16.74 [16.08, 17.49]	0.020
Concurrent ASMs, median [IQR]	1.00 [0.00, 1.50]	3.00 [2.00, 4.00]	3.00 [2.00, 3.00]	<0.001
WBC, median [IQR], ×10^9^/L	6.27 [5.76, 7.08]	6.46 [5.93, 7.73]	6.05 [5.24, 6.62]	0.163
Neutrophil ratio, mean (SD), %	44.34 (7.85)	49.47 (8.13)	48.97 (5.60)	<0.001
Lymphocyte ratio, mean (SD), %	45.66 (8.60)	40.22 (7.63)	39.82 (4.59)	<0.001
Platelets, median [IQR], ×10^9^/L	228.00 [215.50, 248.00]	243.00 [201.00, 293.00]	218.00 [204.00, 286.00]	0.139
CRP, median [IQR], mg/dL	0.06 [0.05, 0.08]	0.10 [0.08, 0.13]	0.16 [0.10, 0.18]	<0.001
NLR, median [IQR]	0.99 [0.78, 1.18]	1.21 [1.00, 1.55]	1.20 [1.00, 1.41]	<0.001
SII, median [IQR]	219.47 [177.56, 293.35]	298.50 [218.28, 382.31]	321.05 [218.54, 395.09]	<0.001

Data are presented as median [interquartile range, IQR] for skewed continuous variables, mean (standard deviation) for normally distributed variables, or *n* (%). *p*-values represent comparisons between the SeLECTS and DRE groups via the Mann–Whitney *U* test, Student’s *t*-test, or chi-square test as appropriate. To eliminate potential hematological confounding from long-term polytherapy, inflammatory indices (WBC, CRP, NLR, SII, and cell ratios) were derived from peripheral blood samples collected at the initial hospital visit prior to extensive drug exposure. Conversely, the number of concurrent ASMs reflects the maximum drug burden recorded at the final follow-up, serving as an indicator of clinical refractoriness. SeLECTS, self-limited epilepsy with centrotemporal spikes; DRE, drug-resistant epilepsy; DS, Dravet syndrome; ASMs, anti-seizure medications.

**Table 2:** Multivariable logistic regression analysis for predictors of refractory epilepsy.

Variable	OR (95% CI)	*p*-value
Age at onset (per year)	0.55 (0.44–0.67)	<0.001
Male (vs. female)	0.56 (0.18–1.64)	0.299
BMI	0.95 (0.82–1.11)	0.498
NLR (per 1-SD increase)	1.61 (1.00–2.80)	0.064
SII (per 1-SD increase)	1.72 (1.01–3.27)	0.068
CRP (per 1-SD increase)	2.79 (1.23–7.43)	0.025

Multivariable logistic regression models were utilized to evaluate the independent association between inflammatory markers and the risk of refractory epilepsy. The odds ratios (ORs) for NLR, SII, and CRP correspond to a 1-standard deviation (SD) increase in these continuous variables. Each inflammatory marker was modeled separately to prevent multicollinearity, with all models adjusting for age at onset, sex, and body mass index (BMI).

### 3.4 Hyperactivation of the JAK-STAT3 pathway in *Scn1a*^+/−^ mice

**Figure 4:**
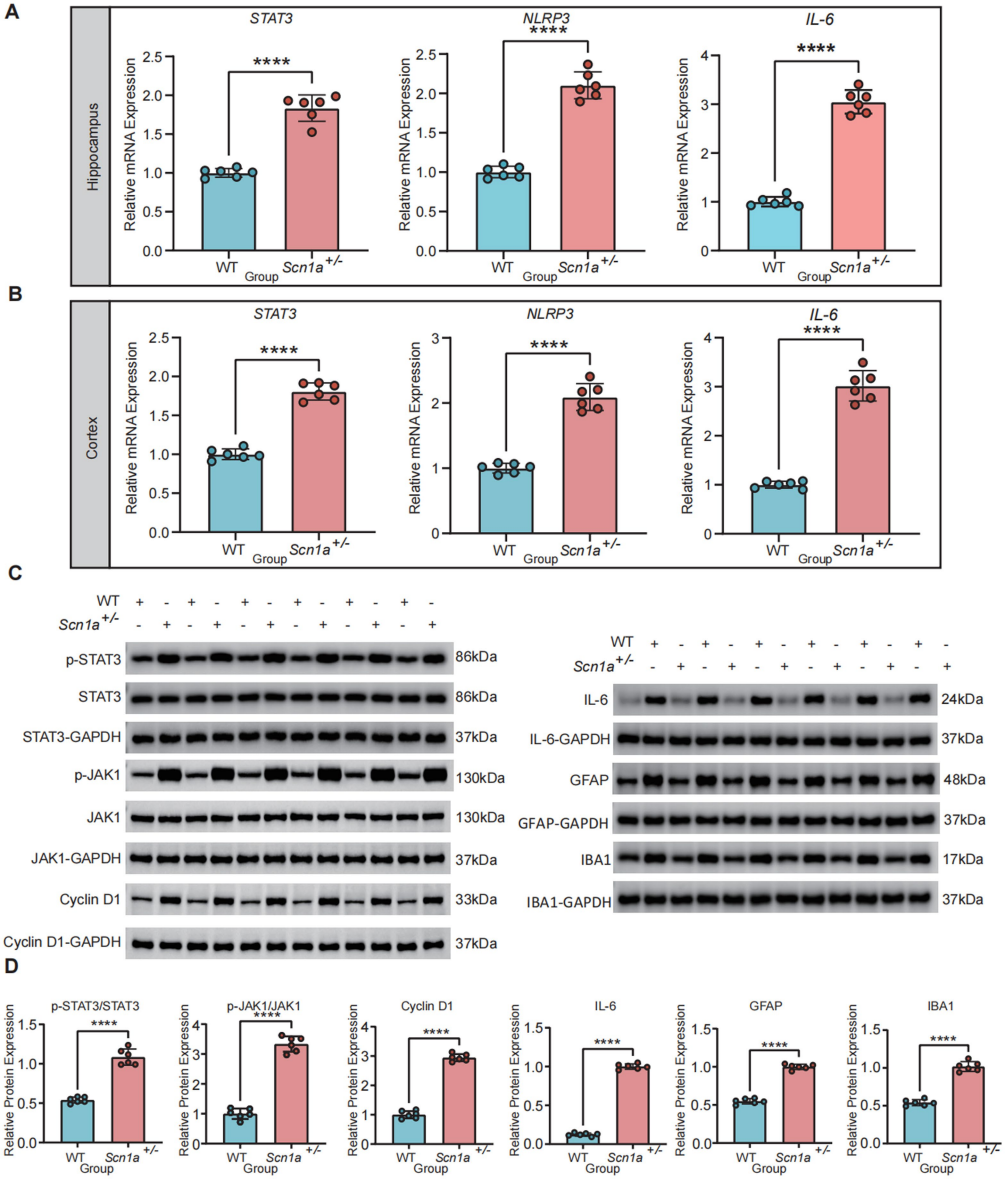
Activation of the JAK-STAT3 signaling axis and neuroinflammatory response in *Scn1a*^+/−^ mice. **(A,B)** Quantitative PCR analysis of *Stat3*, *Nlrp3*, and *Il6* mRNA levels in the hippocampus **(A)** and cortex **(B)** of WT and *Scn1a*^+/−^ mice. **(C)** Representative western blot images showing expression levels of p-STAT3, STAT3, p-JAK1, JAK1, Cyclin D1, IL-6, IBA1, and GFAP in brain tissues. GAPDH served as the loading control. **(D)** Densitometric quantification of relative protein abundance for p-STAT3/STAT3, p-JAK1/JAK1, Cyclin D1, IL-6, GFAP, and IBA1. Data are presented as mean ± SD (^****^p < 0.0001).

To validate the phenotypic susceptibility of our model, the F1 cohorts were subjected to a hyperthermia-induced seizure protocol. Kaplan–Meier survival analysis demonstrated that all *Scn1a*^+/−^ mice exhibited generalized tonic–clonic seizures (equivalent to Racine score 5) at a significantly lower temperature threshold (mean = 41.68 °C; range = 40.5–42.5 °C). Notably, none of the WT littermates exhibited any seizure activity up to the 43.0 °C cutoff limit (Supplementary Figure S2D). Following phenotypic validation, mechanistic verification in the hippocampus and cortex of *Scn1a*^+/−^ mice confirmed the pathway’s involvement. Quantitative PCR demonstrated robust transcriptional upregulation of pathway components (*n* = 6/group). *IL-6* mRNA transcripts surged three fold in both brain regions compared to WT littermates (*p* < 0.0001), accompanied by significant elevations in *STAT3* and *NLRP3* (Figures 4A,B). Western blotting revealed a significant elevation in the ratio of phosphorylated STAT3 (Tyr705) to total STAT3 (p-STAT3/Total STAT3) in *Scn1a*^+/−^ mice (*p* < 0.0001), while total STAT3 expression remained constant. Additionally, IL-6 protein levels were significantly higher in the mutant group, as were the levels of the glial markers IBA1 and GFAP (Figures 4C,D).

To provide a more complete characterization of the canonical signaling pathway and determine whether the increased p-STAT3 translates into functional transcriptional activation, we further evaluated the upstream kinase and downstream targets. Western blot analysis revealed that the ratio of phosphorylated JAK1 to total JAK1 (p-JAK1/Total JAK1) was significantly elevated in the hippocampus of *Scn1a*^+/−^ mice, confirming upstream activation. Additionally, the expression of Cyclin D1, a canonical downstream transcriptional target of STAT3, was also markedly up-regulated compared to WT littermates (Figures 4C,D).

### 3.5 Neuroinflammatory pathology and central-peripheral correlation

**Figure 5:**
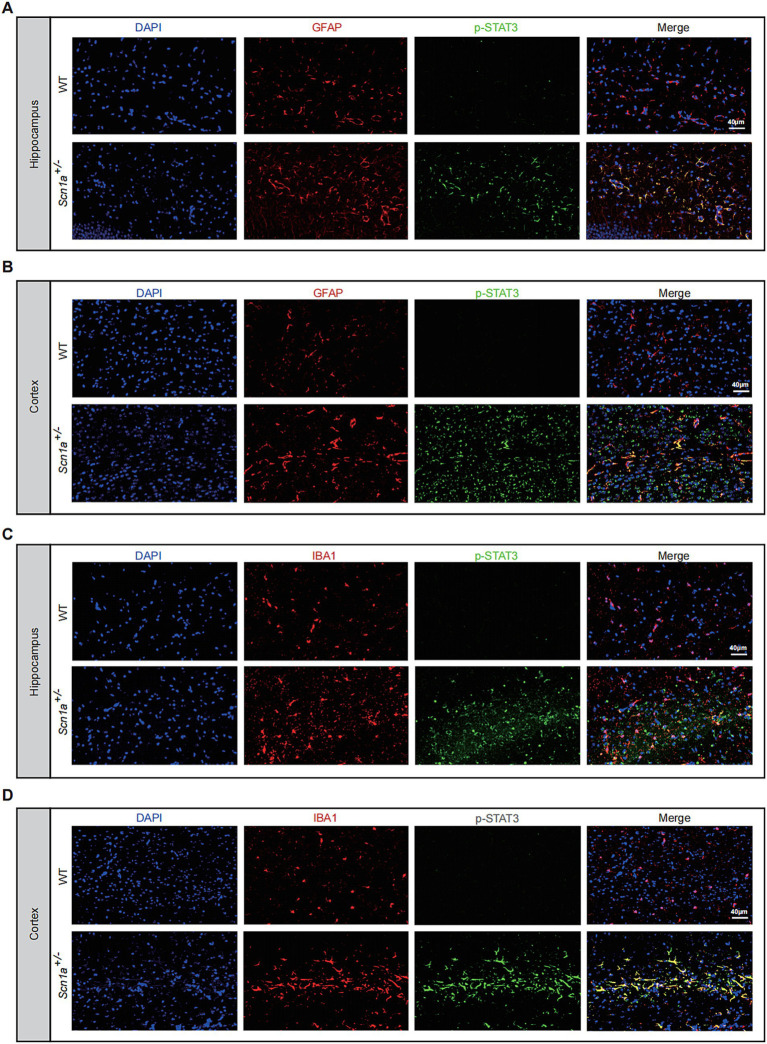
Cellular localization of activated STAT3 in astrocytes and microglia. **(A,B)** Representative double immunofluorescence staining showing co-localization of p-STAT3 (green) with the astrocyte marker GFAP (red) in the hippocampus **(A)** and cortex **(B)** of WT and *Scn1a*^+/−^ mice. **(C,D)** Co-localization of p-STAT3 (green) with the microglial marker IBA1 (red) in the hippocampus **(C)** and cortex **(D)**. Nuclei were counterstained with DAPI (blue). Merged images demonstrate increased p-STAT3 expression and nuclear translocation in the glial cells of *Scn1a*^+/−^ mice. Scale bar = 40 μm.

**Figure 6:**
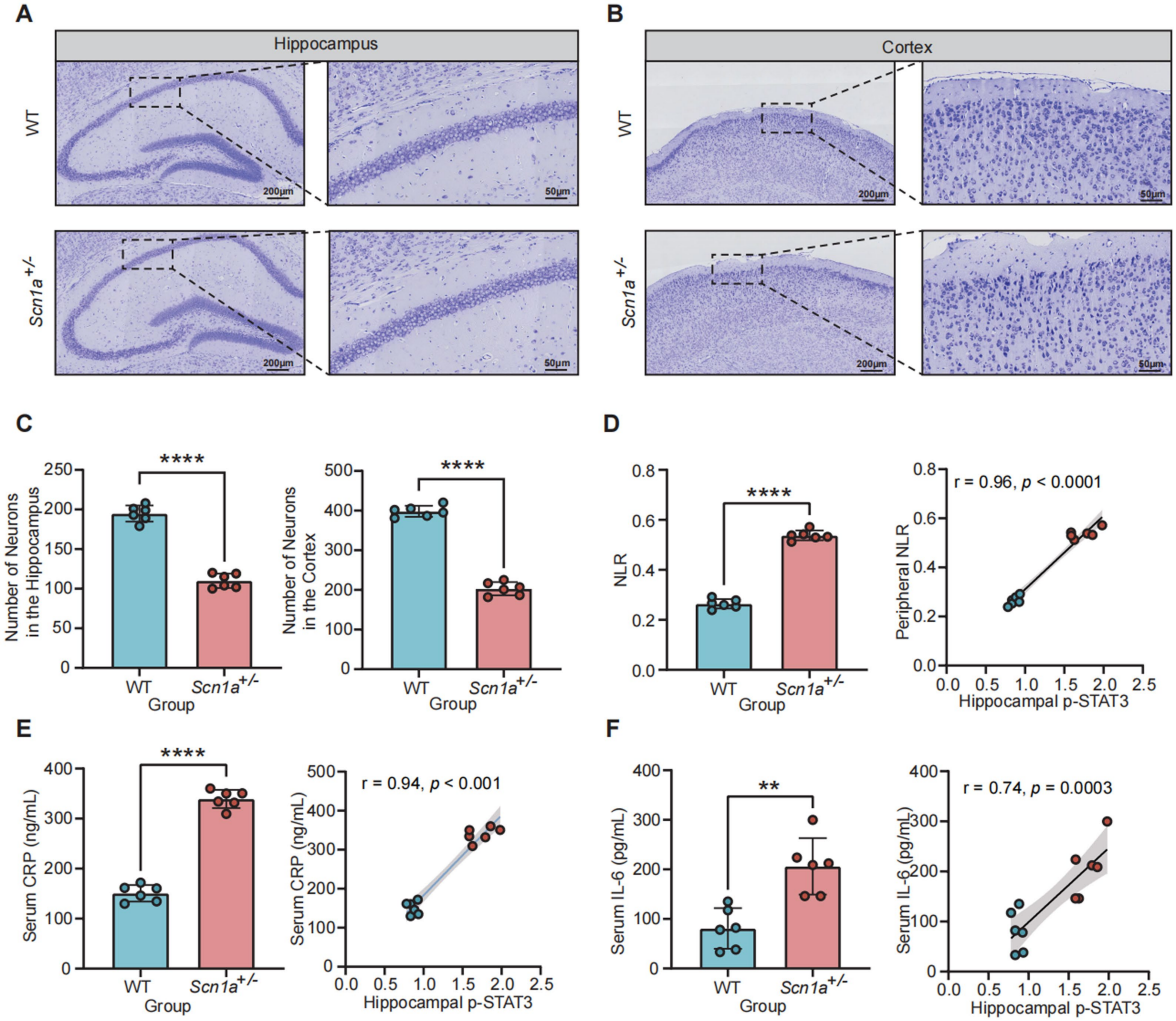
Central neuronal loss and its correlation with systemic inflammatory markers. **(A,B)** Representative Nissl staining images of the hippocampus **(A)** and cortex **(B)** in WT and *Scn1a*^+/−^ mice. Scale bars = 200 μm (left) and 50 μm (right). **(C)** Quantitative analysis showing significantly reduced neuronal density in both brain regions of *Scn1a*^+/−^ mice. **(D–F)** Comparison of systemic inflammatory markers (NLR, serum CRP, and serum IL-6) between WT and *Scn1a*^+/−^ groups (left panels), and their respective Pearson correlation analyses with hippocampal p-STAT3 expression (right panels). Data are expressed as mean ± SD (^**^p < 0.01 and ^****^p < 0.0001).

Double immunofluorescence labeling in the brain parenchyma showed that p-STAT3-positive signals were predominantly localized within GFAP-positive astrocytes and IBA1-positive microglia in both the hippocampus and cortex of *Scn1a*^+/−^ mice. Qualitative observation revealed increased staining intensity and altered glial morphology in the mutant group compared to WT controls (Figures 5A–D; Supplementary Figure S3A). Furthermore, to comprehensively evaluate the cellular distribution of STAT3 activation, we performed co-staining with the neuronal marker NeuN. This analysis revealed co-localization of p-STAT3 within NeuN-positive neurons, indicating that STAT3 signaling is concurrently activated within the neuronal population of *Scn1a*^+/−^ mice (Supplementary Figures S3B,C). Nissl staining and subsequent quantitative analysis demonstrated significant structural disorganization and a reduction in neuronal density (*p* < 0.0001; Figures 6A–C).

Systemic inflammatory markers measured in the peripheral blood were significantly elevated in *Scn1a*^+/−^ mice compared to WT littermates. The blood NLR increased from 0.26 ± 0.02 to 0.54 ± 0.02 (*p* < 0.0001; Figure 6D). Serum CRP levels reached 339.4 ± 18.4 ng/mL in the mutant group compared to 150.7 ± 16.4 ng/mL in WT (*p* < 0.0001; Figure 6E), while serum IL-6 levels rose from 80.8 ± 41.5 pg/mL to 206.2 ± 57.9 pg/mL (*p* < 0.01; Figure 6F). Correlation analyses demonstrated a positive linear association between hippocampal p-STAT3 expression and these peripheral markers, with Pearson correlation coefficients of *r* = 0.96 for NLR (*p* < 0.0001; Figure 6D), *r* = 0.94 for serum CRP (*p* < 0.001; Figure 6E), and *r* = 0.74 for serum IL-6 (*p* = 0.0003; Figure 6F). These correlations should be interpreted cautiously given the limited sample size and the use of composite transcriptomic proxies.

## 4 Discussion

While DS is rooted in *SCN1A* haploinsufficiency, this primary genetic defect alone does not fully account for the progressive severity of comorbidities and phenotypic heterogeneity (Doorn et al., 2023; Shabani et al., 2024). Our multi-dimensional analysis delineates a secondary neuropathological phase, positioning the STAT3 signaling axis as a prominent associated pathway of post-seizure neuroinflammation rather than a passive downstream consequence. Through consensus transcription factor enrichment, we identified STAT3 as a robust hub regulator coordinating a distinct immune-associated signature. In the transcriptomic landscape of *Scn1a*^+/−^ mice, this post-seizure central pathology was substantiated by strong linear correlations between *Stat3* expression and brain-intrinsic inflammatory signatures—specifically NLR, SII, and the CRP-surrogate *Ptx3*. Importantly, because both the primary transcriptomic dataset and our *in vivo* models utilized brain tissues harvested following hyperthermia-induced seizures, these molecular signatures represent a synergistic product of the underlying *Scn1a* haploinsufficiency and the acute seizure-induced inflammatory cascade rather than a pure baseline state. Extending this molecular evidence to clinical reality, population-level validation in our real-world pediatric clinical cohort confirmed that the analogous systemic indices with CRP specifically emerging as an independent correlate for the progression to severe, drug-resistant epilepsy phenotypes. These findings suggest that peripheral inflammatory markers may reflect aspects of the underlying neuroimmune state, although this relationship remains indirect.

Our results are consistent with a growing body of evidence implicating immune-related pathways as central components of epileptogenesis (Cheng et al., 2021; Shi et al., 2022). However, by defining the functional engagement of hub genes such as *STAT3* and the inflammasome component NLRP3, our study moves beyond descriptive to propose a more integrated mechanistic model for DS. We propose a potential interaction between seizure activity and inflammatory processes, a concept substantiated by the robust STAT3 phosphorylation and microglial activation observed in DS mouse models (Liu et al., 2023; Hollis and Lukens, 2025; Li et al., 2025). Crucially, the concurrent activation of the upstream kinase JAK1 and the downstream transcriptional target Cyclin D1 confirms that STAT3 operates as a fully functional regulatory node rather than a passive bystander. This inflammatory cascade, likely orchestrated by crucial nodes like STAT3 and NLRP3 known to promote neuronal hyperexcitability and injury, may in turn compromise the blood–brain barrier. Such a breach provides a potential association between our central transcriptomic findings and the systemic inflammation validated at the population level (Hou et al., 2022; Pinzon-Hoyos et al., 2025). This putative reinforcing relationship, where seizures parallel inflammation and inflammation is closely linked to a lowered seizure threshold, offers a powerful explanation for the refractory nature of epilepsy and the progressive cognitive decline characteristic of DS.

Our identification of the JAK1/STAT3 axis in DS aligns with extensive literature implicating this pathway in broader epileptogenesis (Sun et al., 2023). In various acquired epilepsy models, such as pilocarpine-induced status epilepticus, the JAK-STAT cascade is rapidly hyperactivated, driving reactive astrogliosis, microglial proliferation, and subsequent neuroinflammation (Grabenstatter et al., 2013; Hu et al., 2021). Independent preclinical studies have demonstrated that both genetic manipulation and pharmacological inhibition of STAT3 signaling directly attenuate epileptogenesis and mitigate neuroinflammatory responses (Martín-Suárez et al., 2023; Grabenstatter et al., 2013). By contextualizing our associative clinical and transcriptomic data within this established causal framework, our findings structurally support the proposed therapeutic implications. By demonstrating the functional activation of this exact canonical cascade (p-JAK1/p-STAT3/Cyclin D1) in a genetic model of DS, our study expands the current paradigm. It suggests the JAK-STAT pathway may represent a shared molecular feature across both acquired and genetically driven drug-resistant epilepsies.

The observed correlations suggest a potential relationship with disease severity. Previous studies have shown that elevated systemic inflammatory markers predict higher seizure burden and poorer cognitive outcomes in pediatric epilepsy (Nazeri et al., 2022; Balthazard et al., 2025), corroborating the clinical relevance of our integrative data. Moreover, increased CRP levels and neutrophil-based indices have been linked to drug resistance and status epilepticus, supporting the hypothesis that systemic inflammation not only reflects disease severity but is intimately associated with adverse neurological outcomes (Howe et al., 2023; Olivo et al., 2023). Thus, our results support the clinical justification for targeting neuroinflammation as both a correlative indicator and a therapeutic vulnerability. Critically, our identification of the JAK-STAT axis and the NLRP3 inflammasome as prominent signaling nodes suggests that targeted interventions, such as JAK inhibitors or NLRP3 antagonists, warrant future investigation as novel adjunctive therapies for DS.

Validation of transcriptomic findings within our pediatric clinical cohort provided a critical translational bridge between molecular mechanisms and epidemiological evidence. Specifically, our transcriptomic indices utilized *Ptx3* as a brain-intrinsic homolog to surrogate systemic CRP (Scuderi et al., 2024), while *S100a9*, *Fcgr3*, and *Csf3r* represented neutrophil accumulation (Ryckman et al., 2004; Simard et al., 2011; Fraccarollo et al., 2021), and *Lck* and *Zap70* reflected lymphocyte infiltration (Su et al., 2011; Sawada et al., 2018). These selected myeloid and lymphoid gene signatures inherently capture a composite of resident glial activation and potential immune infiltration. We explicitly clarify that these bulk brain transcriptomic signatures do not equate to direct peripheral cell counts, nor do they act as isolated equivalents of peripheral blood ratios. Rather, they provide a conceptual mathematical framework illustrating a severe central neuroinflammatory state. The biological relevance of this central-peripheral axis is supported by established neuroimmune crosstalk mechanisms; severe central neuroinflammation and consequent glial cytokine release can compromise blood–brain barrier (BBB) integrity, subsequently paralleling systemic immune responses and altered peripheral leukocyte profiles (Engelhardt et al., 2017; Vezzani et al., 2019). The parallel elevation of actual hematological NLR, SII, and CRP observed in our *in vivo* models and pediatric clinical cohort supports the concept of this association, suggesting a concurrent systemic immune dysregulation, though direct real-time causality cannot be established due to temporal sampling differences. This parallel relationship supports a potential clinical rationale for using these accessible peripheral indices to gauge the severity of the underlying neuroimmune pathology. Elevated NLR, CRP, and SII have been widely implicated across a spectrum of neurological disorders. For instance, NLR and CRP levels are significantly higher in Alzheimer’s disease and mild cognitive impairment (Shen et al., 2019; Aprile et al., 2025); higher NLR and SII correlate with white matter hyperintensities in cerebral small vessel disease (CSVD) (Nam et al., 2017; Nam et al., 2022); and SII positively tracks with stroke severity (Liu et al., 2025). These findings suggest that these indices reflect a convergent systemic immune activation across multiple neurological conditions. To evaluate the discriminative capacity of these markers in our cohort, ROC analysis demonstrated that a combined inflammatory panel shows a moderate capacity to distinguish the severe DRE phenotype from SeLECTS (AUC = 0.81). Given the modest effect sizes observed, these systemic indices should be interpreted as correlative indicators reflecting the underlying neuroimmune burden rather than standalone predictive tools. Crucially, by utilizing a pediatric cohort directly comparing DRE, including DS against SeLECTS, we demonstrate that these inflammatory processes are closely linked to disease severity rather than just being a general feature of all epilepsies. From a clinical perspective, NLR, CRP and SII are cost-effective, widely available laboratory parameters that reflect the underlying neuroimmune burden, thereby complementing genetic and imaging evaluations in high-risk patients with pediatric drug-resistant epilepsy, including DS.

The strengths of this study lie in its convergent cross-species framework, which bridges transcriptomic mechanism discovery with population-level clinical validation. By integrating multi-database TF enrichment to identify the *Stat3* hub (Zha, 2025) and validating its correlation with accessible biomarkers in both *Scn1a*^+/−^ mice and our pediatric clinical cohort, we provide a robust translational evidence chain. However, certain limitations warrant consideration. First, the transcriptomic sample size was relatively limited (*n* = 6). We explicitly acknowledge that with such a small cohort, principal component separation might be overestimated, and correlation analyses are inherently sensitive to individual sample variations. While our application of robust linear models (RLM) was intended to statistically mitigate the influence of potential outliers, it cannot fully eliminate the potential for correlation overestimation inherent in small datasets, thus these coefficients should be viewed as indicative of biological trends rather than absolute values. To provide supplementary context, we incorporated a small iPSC-derived dataset (GSE289689) supporting STAT3 hyperactivation. However, due to its limited sample size and biological divergence from our *in vivo* model, these transcriptomic results must be interpreted cautiously, relying primarily on our subsequent in *vivo* and clinical corroboration. Second, the transcriptomic inflammatory indices calculated from bulk tissue lack single-cell resolution, precluding the precise differentiation between resident microglial activation and actual peripheral leukocyte infiltration. These mathematical surrogates must be interpreted cautiously as conceptual representations of the central inflammatory microenvironment rather than direct equivalents of systemic hematological cell ratios. Third, our in *vivo* experiments were exclusively conducted using male mice to minimize the confounding effects of the estrous cycle on baseline neuroimmune states and seizure thresholds. Given the established sex-specific dimorphisms in immune signaling and epilepsy susceptibility, future studies incorporating female cohorts are necessary to ensure broad generalizability. Fourth, while our clinical cohort included specific DS cases, the DS subgroup was relatively small (*n* = 9) within the broader DRE population. Thus, our clinical findings more accurately reflect pediatric drug-resistant epilepsy in general. Furthermore, as this cohort was derived from a single center and lacked systematic control for unmeasured clinical confounders including precise pre-hospitalization seizure burden and acute rescue medications, future multi-center prospective studies are needed to further validate these biomarkers. Finally, definitive causal relationships cannot be inferred from this observational design. Specifically, the temporal misalignment between baseline peripheral blood sampling and post-seizure brain tissue collection precludes establishing a synchronized central-peripheral axis. While the current study does not include direct manipulations to demonstrate whether STAT3 modulation definitively alters seizure phenotypes within our specific model, prior studies employing targeted genetic manipulation of STAT3 have clearly established a causal contribution of this pathway to epileptogenesis (Tipton et al., 2023). Together with existing pharmacologic evidence, these genetic studies strongly support our hypothesis and further contextualize our findings, positioning the STAT3 signaling hub as a critical driver of secondary neuroimmune pathology. Future investigations should employ prospective longitudinal designs combining single-cell transcriptomics to dissect cell-type-specific JAK-STAT3 contributions.

## 5 Conclusion

This study establishes a novel multi-dimensional framework linking molecular inflammation to epilepsy risk, effectively bridging the gap between pathogenic mechanisms in Dravet syndrome and real-world evidence from an assembled pediatric clinical cohort. Our findings suggest that immune dysregulation may be an important associated feature of DS pathophysiology, associated with the hyperactivation of the canonical JAK1/STAT3 signaling cascade and its downstream transcriptional programs. The identification of this axis as a prominent associative feature of severe neuroimmune dysregulation suggests STAT3 as a promising potential therapeutic candidate for disease modification, warranting rigorous functional and interventional validation. Coupled with the validation of NLR, SII, and CRP as accessible clinical indicators of disease severity in pediatric drug-resistant epilepsy, our findings underscore an immediate translational utility. Collectively, these data provide preliminary translational support and a rationale for further investigation of targeted adjunctive therapies mitigating neuroinflammation for both DS and the broader spectrum of DRE.

## Data Availability

Publicly available datasets analyzed in this study are found in the Gene Expression Omnibus under accession numbers GSE112627 and GSE289689. The original raw experimental data and de-identified clinical data supporting the conclusions of this article are available from the corresponding author upon reasonable request, subject to ethical approvals and patient privacy regulations where applicable.

## Supplementary material

SUPPLEMENTARY FIGURE S1 Flowchart detailing the patient screening and enrollment process for the pediatric clinical cohort. Retrospective screening was performed on pediatric inpatients admitted between 2010 and 2025. Patients with self-limited epilepsy with centrotemporal spikes (SeLECTS) and drug-resistant epilepsy (DRE) were sequentially evaluated against stringent inclusion and exclusion criteria. Exclusions were made for duplicate records, incomplete serological data, acute systemic infections at baseline, prior exposure to systemic glucocorticoids or ACTH within 4 weeks, and preexisting hematological or autoimmune disorders. The final cohort analyzed in this study comprised 71 patients with SeLECTS and 69 patients with DRE (specifically including a subgroup of 9 confirmed cases of Dravet syndrome).

SUPPLEMENTARY FIGURE S2 External transcriptomic support, mouse genotyping, and seizure susceptibility. **(A)** Box plot of STAT3 expression (VST normalized counts) in normal and severe epilepsy phenotypes from the independent external dataset GSE289689. **(B)** Gene set enrichment analysis (GSEA) demonstrating significant positive enrichment of the JAK-STAT signaling pathway in the severe phenotype group (NES = 1.53, *p* < 0.01). **(C)** Representative agarose gel electrophoresis image for PCR-based genotyping of mouse tail DNA. Lanes labeled (+/+) represent wild-type mice, and (+/−) represent heterozygous mutant (*Scn1a^tm1Kea^*) mice. **(D)** Kaplan–Meier survival curve illustrating the core body temperature threshold for hyperthermia-induced seizures in WT and *Scn1a*^+/−^ mice (*p* < 0.001).

SUPPLEMENTARY FIGURE S3 Quantitative analysis of immunofluorescence and neuronal localization of p-STAT3. **(A)** Quantitative analysis of the relative fluorescence intensities of p-STAT3, GFAP, and IBA1 in the hippocampus and cortex, corresponding to the representative images in Figure 5. **(B)** Representative double immunofluorescence images showing the co-localization of p-STAT3 (green) with the neuronal marker NeuN (red) in the hippocampus and cortex of WT and *Scn1a*^+/−^ mice. Nuclei were counterstained with DAPI (blue). Scale bars: 40 μm. **(C)** Quantitative analysis of the relative fluorescence intensities of p-STAT3 and NeuN within the neuronal assessment fields. Data are expressed as mean ± SD (^****^*p* < 0.0001, determined by Student’s *t*-test).
