# Supplementary material for: STAT3 signaling is associated with neuroimmune dysregulation in a Dravet syndrome model and pediatric drug-resistant epilepsy
Source: Front Neurosci. 2026 Apr 13;20:1810088. doi: 10.3389/fnins.2026.1810088 (PMC13112372; doi:10.3389/fnins.2026.1810088)
Supplement: Supplementary file 1 [file Table_1.DOCX]

**Supplementary Material**

**Supplementary Methods**

**External Validation via iPSC-Derived Neurons**

To validate the hub regulator identification, the GSE289689 dataset was retrieved from the Gene Omnibus database. This cohort utilized induced pluripotent stem cell (iPSC)-derived neurons from siblings carrying the *SCN1A* (c.434T>C) mutation, stratified into Normal (n = 6) and Severe (n = 3) groups based on clinical phenotype. Raw gene counts were processed using R software (v4.2.1). Duplicate gene symbols were consolidated by summation and low-abundance transcripts with counts less than 10 in fewer than three samples were excluded to minimize noise.

**Differential Expression and Variance Stabilization**

The DESeq2 package (v1.38.0) was employed to perform differential expression analysis via a negative binomial generalized linear model. Variance Stabilizing Transformation (VST) was applied to raw counts to address heteroscedasticity and mitigate the influence of outliers. Significant differentially expressed genes were defined by a False Discovery Rate < 0.05 and an absolute log2 fold change ≥ 0.585.

**Gene Set Enrichment Analysis**

Functional enrichment of the JAK-STAT signaling cascade was evaluated using GSEA. Gene symbols were mapped to Entrez IDs using the org.Hs.eg.db package. The record with the maximum absolute Wald statistic was retained for genes with multiple mappings. All genes were ranked in descending order based on Wald statistics to integrate magnitude and statistical precision. The pre-ranked list was analyzed against the KEGG database using the clusterProfiler package. Statistical significance for the JAK-STAT pathway (hsa04630) was established at *p* < 0.05.

**Animal Husbandry and Hyperthermia Protocol Details Animal Husbandry**

All animals were housed in a specific pathogen-free (SPF) facility under controlled conditions (20–26°C, 40–60% humidity, 12-hour light-dark cycle). Pelleted feed, water, and bedding were sterilized, and mice had ad libitum access to food and water.

**Hyperthermia Induction Protocol**

Prior to testing, mice were acclimated to the rectal temperature probe for 5 min. Heat induction was performed using an infrared lamp. Upon seizure detection or reaching the 43°C safety limit, heating was immediately terminated, and mice were placed on a cooled metal surface to rapidly restore body temperature to approximately 37°C. Seizure severity was scored according to the modified Racine scale: stage 1, mouth and facial movements; stage 2, head nodding; stage 3, forelimb clonus; stage 4, rearing; and stage 5, rearing and falling with generalized tonic-clonic seizures.

**Supplementary Table**

| **Table S1. Summary of sample information for the GSE112627 transcriptomic dataset.** | | | | | | | | |
| --- | --- | --- | --- | --- | --- | --- | --- | --- |
| **GEO accession** | **GSM ID** | **Platform** | **Experiment type** | **Organism** | **Strain** | **Genotype** | **Sample** | **Tissue** |
| GSE112627 | GSM3074527 | Illumina HiSeq 4000 (Mus musculus) | Expression profiling by high throughput sequencing | *Mus musculus* | [129S6/SvEvTac x C57BL/6]F1 | Scn1a+/- | 1: P24_F1_KO_Sz | Hippocampus |
| GSE112627 | GSM3074528 | Illumina HiSeq 4000 (Mus musculus) | Expression profiling by high throughput sequencing | *Mus musculus* | [129S6/SvEvTac x C57BL/6]F1 | Scn1a+/- | 2: P24_F1_KO_Sz | Hippocampus |
| GSE112627 | GSM3074529 | Illumina HiSeq 4000 (Mus musculus) | Expression profiling by high throughput sequencing | *Mus musculus* | [129S6/SvEvTac x C57BL/6]F1 | Scn1a+/- | 3: P24_F1_KO_Sz | Hippocampus |
| GSE112627 | GSM3074532 | Illumina HiSeq 4000 (Mus musculus) | Expression profiling by high throughput sequencing | *Mus musculus* | [129S6/SvEvTac x C57BL/6]F1 | Wild type | 6: P24_F1_WT | Hippocampus |
| GSE112627 | GSM3074533 | Illumina HiSeq 4000 (Mus musculus) | Expression profiling by high throughput sequencing | *Mus musculus* | [129S6/SvEvTac x C57BL/6]F1 | Wild type | 7: P24_F1_WT | Hippocampus |
| GSE112627 | GSM3074534 | Illumina HiSeq 4000 (Mus musculus) | Expression profiling by high throughput sequencing | *Mus musculus* | [129S6/SvEvTac x C57BL/6]F1 | Wild type | 8: P24_F1_WT | Hippocampus |

**Table S2. Primer sequences used for genotyping and qRT-PCR analysis.**

| **Target Gene / Purpose** | **Primer Name** | **Sequence (5'–3')** |
| --- | --- | --- |
| **Genotyping** |  |  |
| *Scn1a* | Common | AGTCTGTACCAGGCAGAGAACTTG |
|  | WT-R | CCCTGAGATGTGGGGTGAATAG |
|  | Mut-R | AGACTGCCTTGGGAAAAGCG |
| **qRT-PCR** |  |  |
| *STAT3* | Forward | TGCGGAGAAGCATTGTGAGT |
|  | Reverse | CCAGTTTTCCAGACGGTCCA |
| *IL-6* | Forward | GTCCTTCCTACCCCAATTTCCA |
|  | Reverse | TAACGCACTAGGTTTGCCGA |
| *NLRP3* | Forward | GTACCCAAGGCTGCTATCTGG |
|  | Reverse | GGACACTCGTCATCTTCAGCA |
| *GAPDH* | Forward | AGGTCGGTGTGAACGGATTTG |
|  | Reverse | GGGGTCGTTGATGGCAACA |
