# Supplementary figures and images for: STAT3 signaling is associated with neuroimmune dysregulation in a Dravet syndrome model and pediatric drug-resistant epilepsy
Source: Front Neurosci. 2026 Apr 13;20:1810088. doi: 10.3389/fnins.2026.1810088 (PMC13112372; doi:10.3389/fnins.2026.1810088)

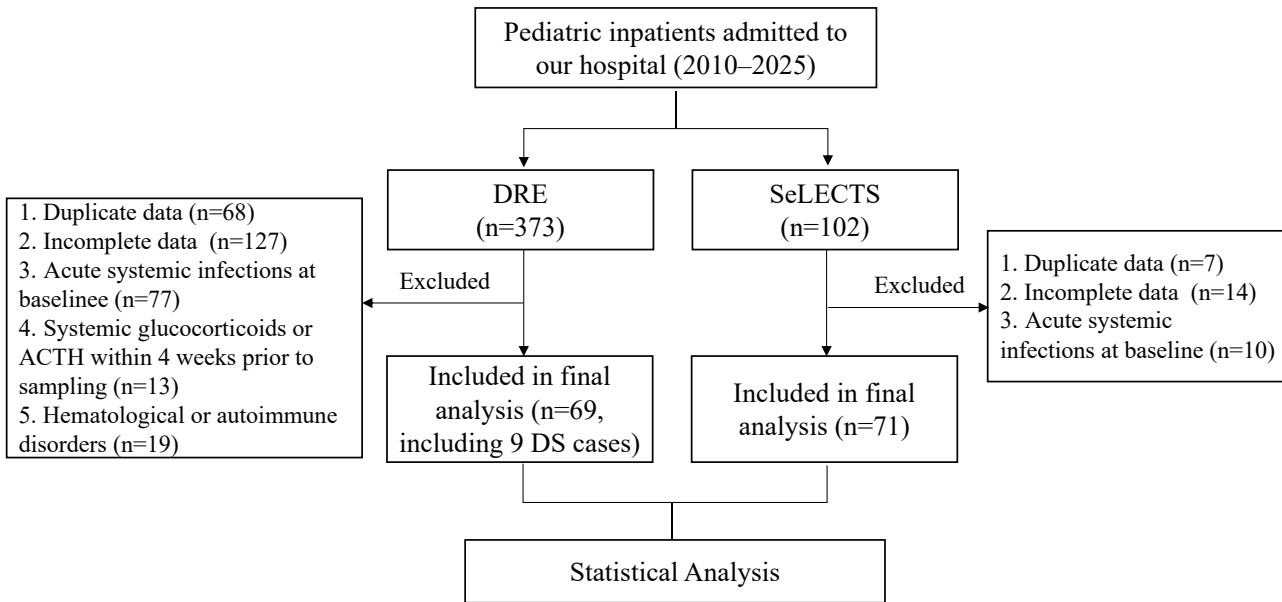

Supplement: SUPPLEMENTARY FIGURE S1 — Flowchart detailing the patient screening and enrollment process for the pediatric clinical cohort. Retrospective screening was performed on pediatric inpatients admitted between 2010 and 2025. Patients with self-limited epilepsy with centrotemporal spikes (SeLECTS) and drug-resistant epilepsy (DRE) were sequentially evaluated against stringent inclusion and exclusion criteria. Exclusions were made for duplicate records, incomplete serological data, acute systemic infections at baseline, prior exposure to systemic glucocorticoids or ACTH within 4 weeks, and preexisting hematological or autoimmune disorders. The final cohort analyzed in this study comprised 71 patients with SeLECTS and 69 patients with DRE (specifically including a subgroup of 9 confirmed cases of Dravet syndrome). [file Image_1.PDF]

**A**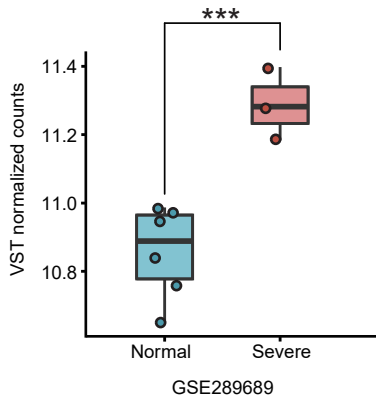**B**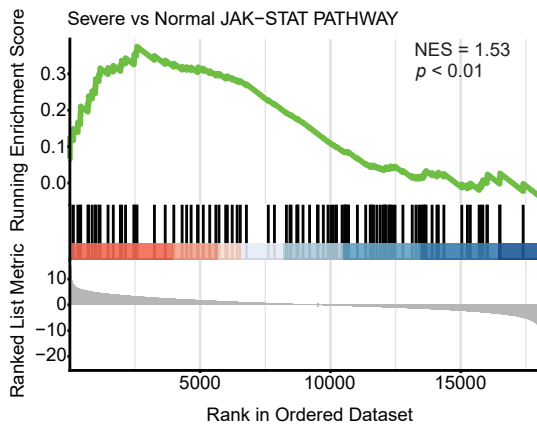**C**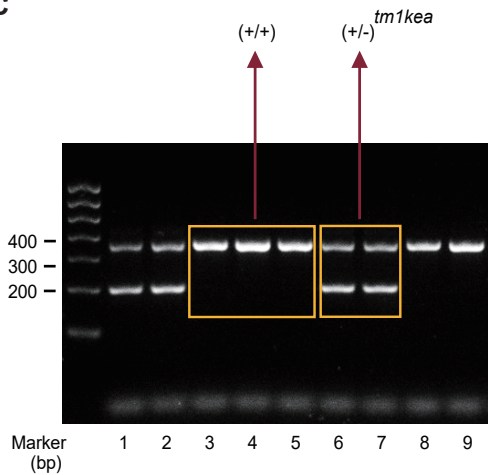**D**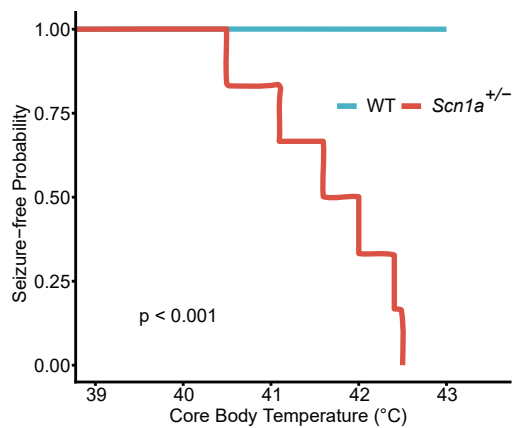

Supplement: SUPPLEMENTARY FIGURE S2 — External transcriptomic support, mouse genotyping, and seizure susceptibility. (A) Box plot of STAT3 expression (VST normalized counts) in normal and severe epilepsy phenotypes from the independent external dataset GSE289689. (B) Gene set enrichment analysis (GSEA) demonstrating significant positive enrichment of the JAK-STAT signaling pathway in the severe phenotype group (NES = 1.53, p < 0.01). (C) Representative agarose gel electrophoresis image for PCR-based genotyping of mouse tail DNA. Lanes labeled (+/+) represent wild-type mice, and (+/−) represent heterozygous mutant (Scn1atm1Kea) mice. (D) Kaplan–Meier survival curve illustrating the core body temperature threshold for hyperthermia-induced seizures in WT and Scn1a+/− mice (p < 0.001). [file Image_2.PDF]

**A**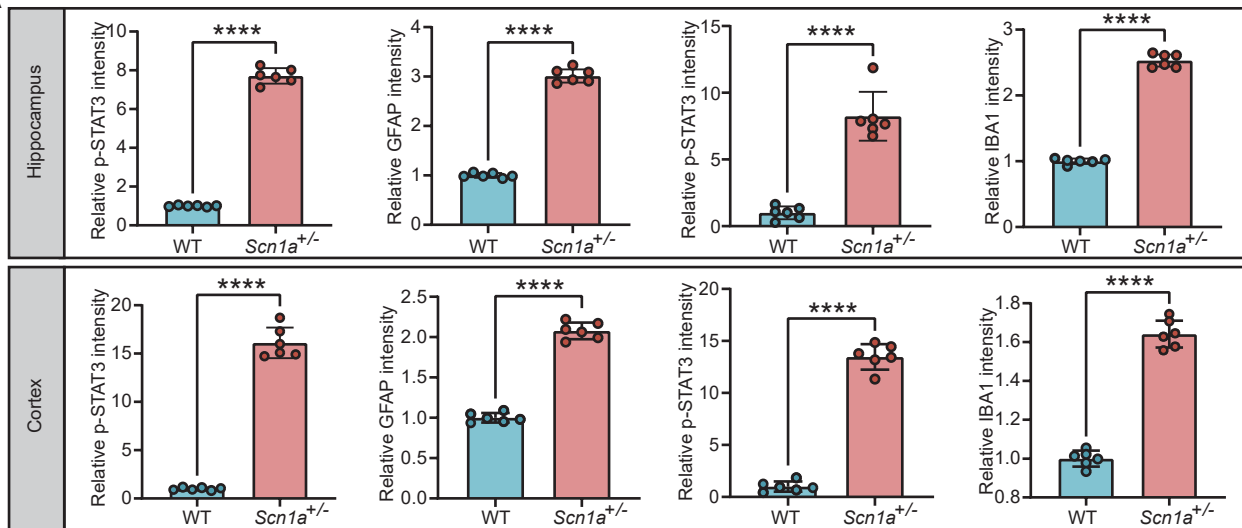**B**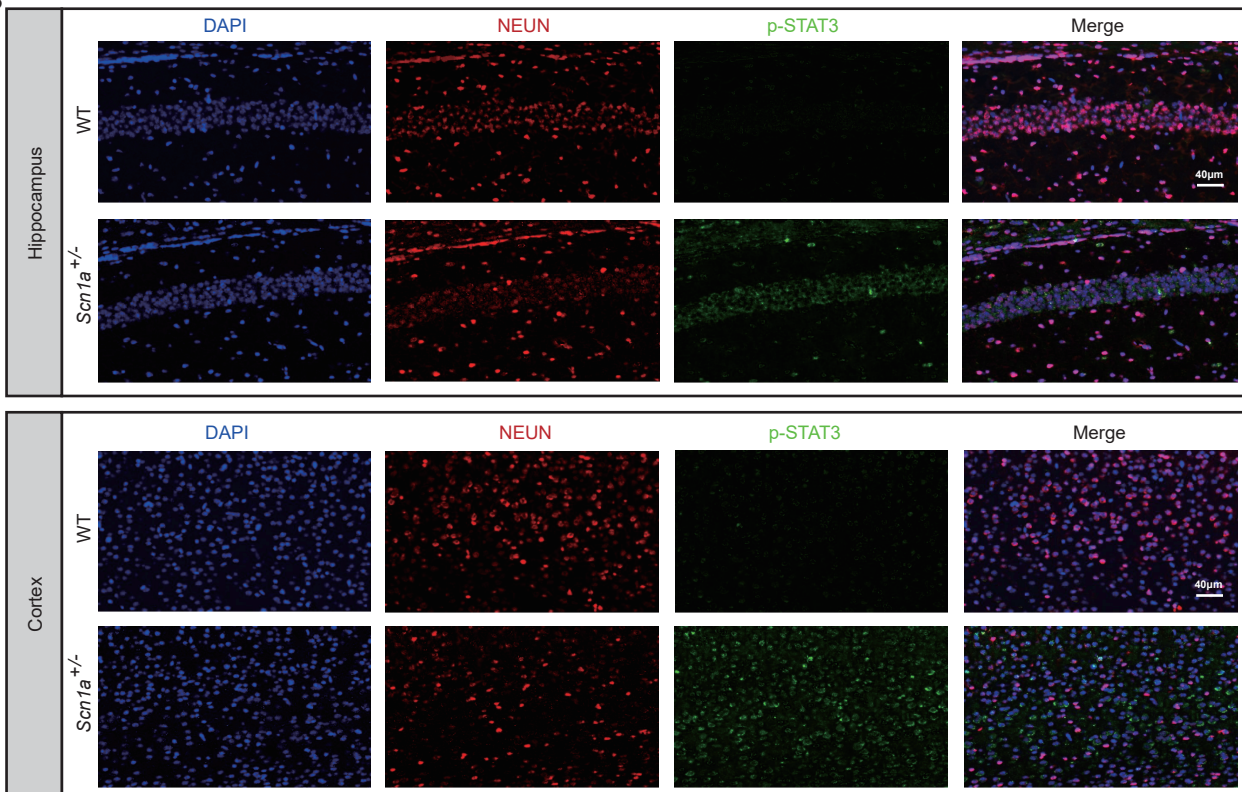**C**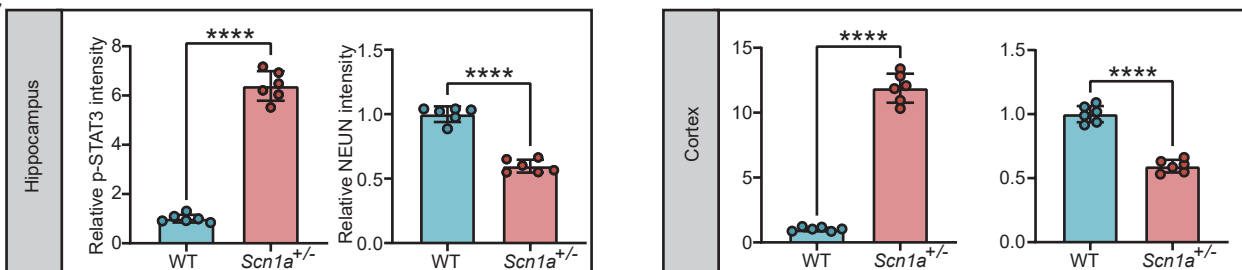

Supplement: SUPPLEMENTARY FIGURE S3 — Quantitative analysis of immunofluorescence and neuronal localization of p-STAT3. (A) Quantitative analysis of the relative fluorescence intensities of p-STAT3, GFAP, and IBA1 in the hippocampus and cortex, corresponding to the representative images in Figure 5. (B) Representative double immunofluorescence images showing the co-localization of p-STAT3 (green) with the neuronal marker NeuN (red) in the hippocampus and cortex of WT and Scn1a+/− mice. Nuclei were counterstained with DAPI (blue). Scale bars: 40 μm. (C) Quantitative analysis of the relative fluorescence intensities of p-STAT3 and NeuN within the neuronal assessment fields. Data are expressed as mean ± SD (****p < 0.0001, determined by Student’s t-test). [file Image_3.PDF]
